# A versatile open-source analysis of the limiting efficiency of photo electrochemical water-splitting

**DOI:** 10.1038/s41598-018-30959-9

**Published:** 2018-08-24

**Authors:** Isaac Holmes-Gentle, Klaus Hellgardt

**Affiliations:** Department of Chemical Engineering, Imperial College London, London, SW7 2AZ UK

## Abstract

Understanding the fundamental thermodynamic limits of photo-electrochemical (PEC) water splitting is of great scientific and practical importance. In this work, a ‘detailed balance’ type model of solar quantum energy converters and non-linear circuit analysis is used to calculate the thermodynamic limiting efficiency of various configurations of PEC design. This model is released as freely accessible open-source (GNU GPL v3) code written in MATLAB with a graphical user interface (GUI). The capabilities of the model are demonstrated by simulating selected permutations of PEC design and results are validated against previous literature. This tool will enable solar fuel researchers to easily compare experimental results to theoretical limits to assess its realised performance using the GUI. Furthermore, the code itself is intended to be extendable and so can be modified to include non-ideal losses such as the over-potential required or complex optical phenomena.

## Introduction

Photosynthesis harnesses the solar resource by converting the energy incident on the earth into a storable fuel which has enabled great change in the history of life. A vast quantity of our primary energy use continues to come from fossil fuels^1^, which ultimately derives from ancient photosynthesis. In light of the current social and political pressures to move towards a more immediately sustainable future, there has been much hope in realising an economically feasible ‘artificial photosynthesis’ process capable of meeting the modern energy demand. One such photo-synthetic fuel commonly studied is hydrogen produced through photo-electrochemical reduction of water.

The overall chemical equation for water splitting is found in eq. (1), where the Gibbs energy of reaction *G*° under standard conditions can be expressed as a cell potential ${{E}}_{{cell}}^{^\circ }$ using the equation ${\Delta }{{G}}^{^\circ }=-\,{nF}{{E}}_{{cell}}^{^\circ }$. Here, *n* is the number of electrons transferred per product formed and *F* is the Faraday constant. In an electrochemical process, the oxidation reaction occurs at the anodic interface and is spatially separated by an electronic conductor, from the reduction reaction at cathodic interface. Correspondingly, the standard cell potential is expressed as ${{E}}_{{cell}}^{^\circ }={{E}}_{{cathode}}^{^\circ }-{{E}}_{{anode}}^{^\circ }$ where ${E}_{electrode}^{^\circ }$ is the standard reduction potential of that electrode (cathode/anode).

1 $$2{H}_{2}O\to 2{H}_{2}+{O}_{2},\,\,\,\,\,\,\,\,\,\,{\rm{\Delta }}{G}^{{}^{\circ }}=237\,{\rm{k}}{\rm{J}}\,{{\rm{m}}{\rm{o}}{\rm{l}}}^{-1},\,\,\,\,\,\,\,\,\,\,{E}_{cell}^{{}^{\circ }}=-\,1.23\,{\rm{V}}$$

It can be derived from the Nernst equation for each half reaction $({E}={{E}}^{^\circ }-\frac{{RT}}{{nF}}\,{ln}({K}))$ that both reduction potentials shift by −59 mV per unit pH change. Therefore, regardless of pH, the cell potential of a cell with the cathode and anode in equilibrium (at a partial pressure of 10^5^ Pa) with H_2_ and O_2_, respectively, will be −1.23 V. In reality, kinetic losses will mean a voltage greater than this is required to drive the reaction, and this difference is termed the over-potential, *V*_*o*_ for each cell. As the ideal thermodynamics limits of solar fuel conversion for different configurations is studied here, it is assumed *V*_*o*_ = 0.

Under adiabatic conditions, the total enthalpy of reaction (*ΔH*° = 286 kJ mol^−1^) must be provided as electrical energy. Hence, the thermo-neutral cell potential (the potential at which there is no temperature change under adiabatic conditions) will be −1.48 V. If heat is supplied reversibly from the ambient surroundings at 298.15 K (isothermal), the minimum electrical energy required will therefore be the Gibbs energy of reaction and the minimum cell potential required will be −1.23 V. Therefore, this value will be used in this analysis and for convenience the negative sign will be dropped henceforth: ${{E}}_{{rxn}}=-\,{{E}}_{{cell}}=1.23\,{\rm{V}}$.

A negative cell potential (*i*.*e*. positive Gibbs energy) indicates that this process is not thermodynamically spontaneous, and hence requires free energy in order to drive the reaction. In a solar driven photo-electrochemical cell, this free energy is generated through the excitation of charge carriers in one or more photo-absorbers by absorption of incoming solar photons. The number of photo-absorbers is denoted by *N*_*photo*_, and systems where *N*_*photo*_ = 2 are commonly referred to as tandem PEC cells. Some designs can employ multiple electrolysers and in this case, the total voltage that the photo-absorber(s) are required to produce is therefore ${{V}}_{{total}}={{N}}_{{elec}}({{E}}_{{rxn}}+{{V}}_{{o}})$.

The solar to fuel efficiency for a single electrolyser system is given by ${{\eta }}_{{STF}}=({J}{{\eta }}_{{f}}{{E}}_{{rxn}})/{{P}}_{{solar}}$ where *J* is the current density, *η*_*f*_ is the faradaic efficiency of the fuel producing electrochemical reaction, and *P*_*solar*_ is the power density of the solar spectrum. Consequently, for a water splitting process with a faradaic efficiency of unity, the solar to hydrogen efficiency will be ${{\eta }}_{{STH}}=({J}\times 1.23[{V}])/{{P}}_{{solar}}$. If there are multiple electrochemical cells in series, the current flowing through each will be the same and the solar to hydrogen efficiency will be given by eq. (2), again assuming η_*f*_ = 1.

2 $${{\eta }}_{{STH}}=\frac{{{N}}_{{elec}}{J}\times 1.23[{V}]}{{{P}}_{{solar}}}$$

The efficiency of a directly coupled PV + electrolyser or integrated PEC cell is not simply ${{\eta }}_{{STH}}={{\eta }}_{{PV}}{{\eta }}_{{electrolyser}}$ where η_*PV*_ is the standalone efficiency of the solar cell(s) and ${\eta }_{electrolyser}$ is the efficiency of the electrolyser(s). This is because η_*PV*_ is often stated at maximum power point (mpp), rather than the operating point of electrolysers. A decoupled system that ultilses DC–DC converters, would allow the PV to operate at the mpp whilst maintaining a voltage suitable for electrolysis, though there will be energy losses associated with this voltage conversion.

There has been much work into the fundamental efficiency limits of quantum energy converters and in particular photo-electrochemical energy conversion^2–12^ which is summarised in Table 1. The seminal works are discussed in a book chapter by Bolton *et al*.^4^, which demonstrated the equivalence of many of the historical models. In more recent work, many researchers have investigated practical efficiency limits of solar fuel processes^8–15^ and different optical configurations^9,10^.

**Table 1:** Notable literature on solar fuel conversion.

Year	Reference	Notes
1961	Shockley & Queisser^2^	Laid the foundations of ‘detail balance’ theory for photo-voltaic energy conversion
1977	Ross & Hsiao^3^	Outlined generalised thermodynamics for photochemical solar energy conversion
1981	Bolton *et al*.^4^	A comprehensive review and a demonstration of the equivalence of previous models
1984	Weber and Dignam^5^	Investigates efficiency of single and tandem devices side-by-side and in series configuration
1985	Bolton *et al*.^6^	Proposes a classification system (*e*.*g* S2, D4 *etc*.) and outlines limiting and realisable efficiencies
2006	Hanna & Nozik^7^	Extended analysis to carrier multiplication absorbers
2013	Hu *et al*.^8^	Realisable efficiencies for tandem PEC systems
2014	Seitz *et al*.^9^	Investigates practical efficiencies of PEC systems (up to 2 photo-absorbers) in different optical configurations
2015	Jacobsson *et al*.^10^	Investigates side-by-side configuration for up to 4 photo-absorbers
2016	Fountaine *et al*.^11^	Analysis of both thermodynamic and practical limits compared reported efficiencies
2017	Seger *et al*.^12^	Web-based javascript implementation for tandem PEC systems optically in series

Whilst the models used to calculate the efficiency are often well documented, the method of numerical solution is often less clear and any code used not openly available. Recent work by Seger *et al*.^12^ aims to address this and they maintain a web based efficiency model (www.solarfuelsmodeling.com). This is a javascript implementation for a single or tandem configuration (both electrically and optically in series), which can model both maximum and practical losses. Whilst an undeniably useful tool for the solar fuels community, the configuration is fixed as a two photo-absorber cell, optically and electrically in series. Therefore the solution offered here is unique in its flexibility to model configurations previously understudied.

As explained by Bolton *et al*.^4^, calculation of the photo-electrochemical efficiency assuming no free energy losses is thermodynamically impossible at feasible device and sun temperatures. Intrinsic free energy losses in any heat engine, which includes solar quantum energy converters, are unavoidable^16^. We have reviewed the literature and found numerous examples of calculated photo-electrochemical energy conversion efficiencies which neglect the inescapable radiative emission Fig. 2a^10^,Fig. 2^17,18^, Fig. 7^19^.It is possible that the reason the second law of thermodynamics is neglected, is due to a lack of easily available tools to calculate the limiting photo-electrochemical efficiency.

Here, we outline a methodology and an open-source implementation to calculate the limiting efficiency of various configurations of solar water splitting devices. The script is written in MATLAB, a language common to engineers. Both the script and a stand-alone executable (packaged with the MATLAB Compiler) are provided under an open-source license meaning that it can be modified by researchers to suit their needs. There are various configurations for which the limiting efficiencies have not been well studied, such as those outlined by Brillet *et al*.^20^.

## Overview of model and assumptions

**Figure 1:**
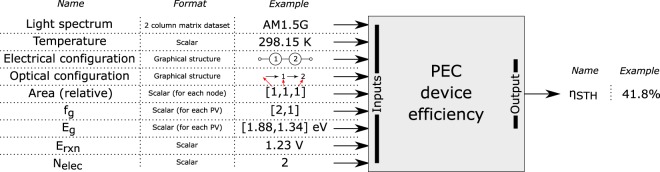
Schematic of model inputs and outputs with example data.

Figure 1 shows an overview of the inputs and outputs of the model, along with example data for the inputs and outputs.

A ‘detailed balance’ approach was taken^7,11,21^, and the following assumptions were made in order to use the standard ideal diode equation: Each cell is planar with an abrupt absorption threshold and complete absorption of above band gap photons. Carrier generation from ambient and from radiative emission from other cells was neglected. Furthermore, one photon excites one electron (*i*.*e* no carrier multiplication). Therefore, the current-voltage relationship of a single photo-absorber is given in eq. (3), where *J* is the current density of the cell, *J*_*L*_ the light induced current, *J*_*o*_ the reverse saturation current, *V* the voltage of the cell, *T*_*a*_ the ambient temperature and *b*_*AM1*.*5G*_ the solar photon flux normal to the surface per energy interval *dE*. The constants *q*, *h*, *c*, *k* are the elementary charge, Planck constant, speed of light and the Boltzmann constant respectively.

3 $$J={J}_{L}-{J}_{o}({e}^{(kV/k{T}_{a})}-1)\,\,{J}_{L}=q{\int }_{{E}_{g}}^{\infty }{b}_{AM1.5G}{dE}\,\,{{J}}_{o}=\frac{2{f}_{g}q\pi }{{h}^{3}{c}^{2}}{\int }_{{E}_{g}}^{\infty }\frac{{{E}}^{2}}{{{e}}^{E{/}k{{T}}_{{a}}}-1}{dE}$$

A geometric factor *f*_*g*_ is specified for each photo-absorber, which is equal to one when the radiative emission is constrained to the front side (*i*.*e* has a perfect back reflector), and equal to two if radiative emission can leave from both front and back sides. Henceforth, *f*_*g*_ = 1 is assumed for all sub-cells except sub-cells optically in series, where unabsorbed light must pass through the photo-absorber to the next (and therefore *f*_*g*_ = 2). Finally, unless otherwise stated, AM1.5 G spectrum (ASTM G173^22^) and an ambient temperature of 298.15 K was used henceforth.

The full derivation of the model and information about the numerical methods used, is outlined in the supplementary information.

## Configurations of water splitting devices

The theoretical limiting efficiency is defined by the conceptual configuration of the photo-electrochemical system. Any PEC configuration can be categorised by: 1) Number of photo-absorbers 2) Electrical configuration 3) Optical configuration.

**Figure 2:**
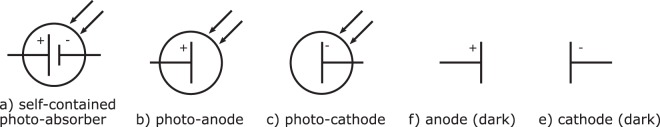
Photo-electrochemical cell components.

The generic photo-electrochemical cell components are shown in Fig. 2, where the self-contained photo-absorber is a solar cell of any type. A photo-electrode is a semiconductor-electrolyte junction and a dark electrode is a metal-electrolyte junction. At each electrode, it must be stated what electrochemical oxidation or reduction process is occurring. As in this work water splitting is investigated, any anode and cathode discussed henceforth, will refer to the oxidation and reduction of water, respectively.

## Electrical configuration

The various components outlined in Fig. 2 can now be electrically connected into a circuit. A complete photo-electrochemical cell circuit must contain at least one cathode, one anode, and one photo-absorber component. In order to match the solar spectrum to the necessary photo-voltage required to split water, photo-absorbers are commonly connected electrically in series. Designs that encompass several electrolysers in series, have demonstrated the current record solar-to-hydrogen efficiency^23^.

## Optical configuration

The optical configuration has a significant impact on the limiting efficiency of the conceptual design^9^ and there will be multiple optical configurations for any conceptual design that has more than one photo-absorber. Light may pass through one photo-absorber and upon to others, allowing for portions of the light spectrum not utilised by the first to be captured by the second. Such a configuration, henceforth, is referred to as *‘optically in series’*. Alternatively, photo-absorbers could be placed *‘optically in parallel’*, where each photo-absorber is placed side-by-side.

In order to represent the optical configuration, a directed graph schematic will be used. Edges indicate the path of the light to nodes which represent each numbered photo-absorber. The first node is the incoming solar spectrum where there is no photo-absorber and so it is unlabelled. For example, a two photo-absorber system could be:

- Optically in series: →1 → 2
- Optically in parallel:

In order to simplify the current schematic, at any split (*i*.*e*. at any node which has 2+ successor nodes), the edge weight defines both the fraction split in the photon flux and the relative area of the subsequent node. In the previous ‘optically in parallel’ example, the edge weight of 1/2 denotes that half the light is transferred to a photo-active area of half the previous node. Currently, optical losses (such as reflection) or spectrum specific optics such as dichroic mirrors are not included in this analysis. Furthermore, it is assumed each photo-absorber has an abrupt absorption threshold and absorbs above bandgap light entirely. As the open-source code is easily extendible, such complex configurations and optical non-idealities could be incorporated in the future.

## Example configurations

**Figure 3:**
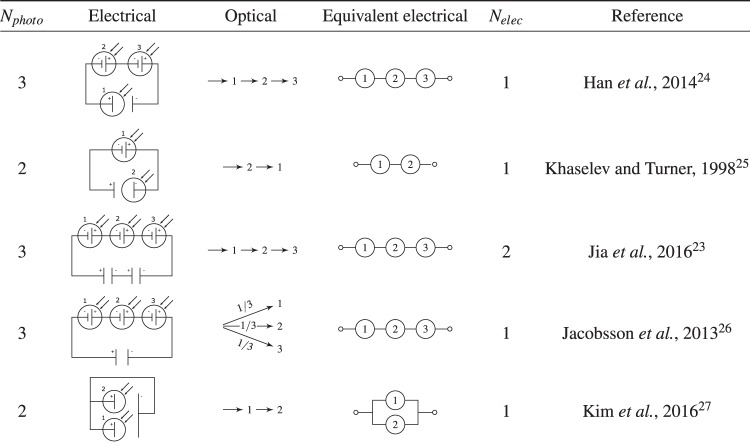
Table of selected example conceptual configurations of various PEC devices.

The table in Fig. 3 shows a number of example configurations of water splitting systems taken from selected literature^23–27^. As demonstrated by Hu *et al*.^8^, the limiting efficiency analysis of PV + electrolysis systems is mathematically equivalent to photo-electrode systems (semiconductor-liquid junctions), and hence Fig. 3 includes the equivalent electrical circuits schematics which will be used henceforth for the theoretical limiting efficiency analysis. In order to save space, the circuit is completed by the number of electrolysers stated. Although uncommon in literature, photo-electrodes could be placed electrically in parallel and optically in series as demonstrated in the work by Kim *et al*.^27^.

## Results

**Figure 4:**
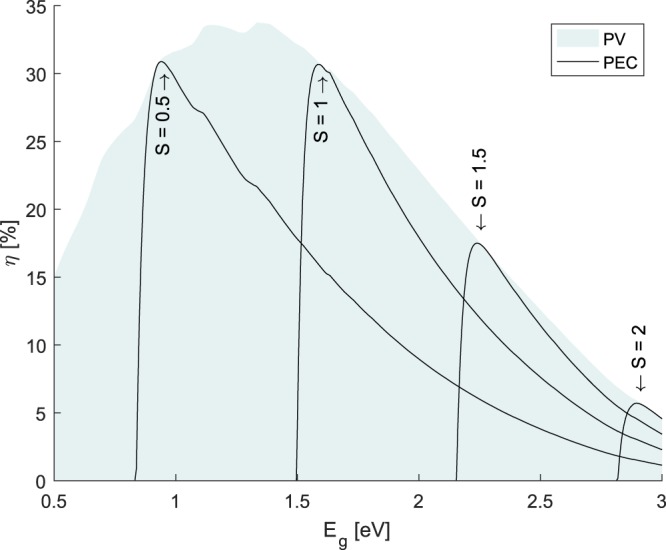
Efficiency of PV (at mpp) and PEC/PV + EC systems for various repeating unit connection ratios, S, under 1 Sun illumination.

As seen in Fig. 4, the PEC efficiency has a distinctive curve which is lower than the PV efficiency (at mpp) at all values except for the point where the maximum power point voltage *V*_*mpp*_ matches with the required voltage to drive the electrochemical cell *V*_*tot*_. At lower bandgaps than this maximum, the system becomes voltage limited. At higher bandgaps, the system provides a greater voltage than required and so this mismatch leads to an efficiency lower than if the voltage is unconstrained and the system can operate at the maximum power point potential.

The capabilities of the code is demonstrated through four examples:

1. Repeating units electrically in series and optically in parallel - Demonstrates that the efficiency of PV + EC systems can be optimised by matching the maximum power point to the load
2. Effect of solar concentration - Demonstrates that for PEC systems under certain conditions, solar concentration can improve the maximum efficiency to a much larger degree than the efficiency improvement for PV alone
3. Permutation of tandem designs - Maximum efficiency versus both bandgaps (*E*_*g*,1_ and *E*_*g*,2_)
4. Global maximum efficiency for selected permutations of design - Bandgap optimisation for greatest η_*STH*_ for various permutations of design.

### Example 1: Repeating units electrically in series and optically in parallel

There is an interesting property for repeating units (of photo-absorber configurations) that are electrically in series and optically in parallel, with each unit getting the same fraction of the total light. It can be shown that all systems with the same ratio of the number of electrochemical cells (*N*_*elec*_) to number of repeating units (*N*_*RU*_) have the same theoretical limiting efficiency. An example of this schematically (blue = optical, black = electrical) is shown in eq. 4 for the case where this ratio is unity.(4)

For integrated photo-electrochemical systems, this repeating unit connection ratio may be constrained inherently by the conceptual design. Essentially, this is as the electrochemical interfaces may be part of the repeating unit, so adjusting *N*_*RU*_ will change *N*_*elec*_. Whereas for separated PV + electrolysis designs, the ratio can be more easily adjusted as the number of photovoltaic cells are independent of the number of electrochemical cells. PV + electrolysis systems can therefore maximise the limiting efficiency by judicious matching of the total photo-voltage produced to the total load of the electrochemical cells. This type of optimisation has been previously discussed and demonstrated, where the operating point of the photovoltaic system is matched with the electrolysis system^14,28–30^.

For the simplest repeating unit () of a single photo absorber (, → 1), we will define the repeating unit connection ratio as $S={N}_{elec}/{N}_{photo}$ as there is one photo-absorber per repeating unit. Figure 4 shows the efficiency of a PEC/PV + EC system versus the PV efficiency at maximum power point. It can be clearly seen efficiency gains can be achieved by matching the load voltage with the operating voltage that maximises the solar cell efficiency. For a photo-anode and (hence *S* is fixed at unity), this method of optimisation will not be available and the maximum efficiency will then be 30.7%. If *S* is allowed to vary, the maximum is 33.8% found at a value of *S* = 0.801 with *E*_*g*_ = 1.34 eV, which approximately corresponds to 5 single photo-absorber repeating units connected to 4 electrolysers. This type of analysis has been extensively discussed by Patel *et al*.^14^, where they investigated repeating unit which consisted of a 1 to 7 junction solar cells that were optically in series.

### Example 2: Effect of solar concentration

**Figure 5:**
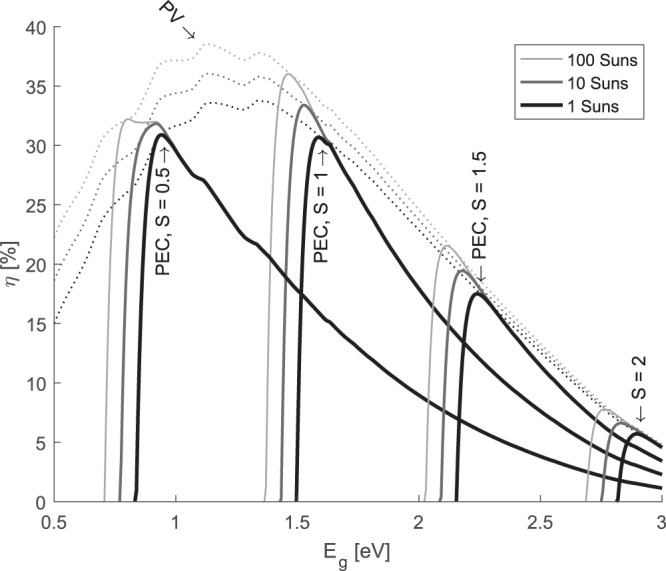
Efficiency of PEC systems for various solar concentrations (1, 10, 100 Suns) and various repeating unit connection ratios (S = 0.5, 1, 1.5, 2) under 1 Sun illumination.

Solar concentration has a significant effect on the limiting efficiency^31^, as seen in Fig. 5. As this increases the open circuit voltage, it can allow the PEC system to operate with smaller bandgaps whilst still driving the electrochemical reaction. If the PV efficiency is higher at smaller bandgaps, as for the example of *S* = 1, higher solar concentration leads to significantly higher efficiencies due to better voltage matching. On the contrary, if the PV efficiency is lower at smaller bandgaps, such as the case for S = 0.5, the efficiency only marginally improves with solar concentration. Further analysis of this is required that takes into account that concentrating optics have an acceptance solid angle and so cannot use the full diffuse spectrum accounted for in AM1.5G. However, this simple analysis demonstrates the importance of solar concentration on the theoretical limiting efficiency of photo-electrochemical water splitting.

### Example 3: Permutation of tandem designs

**Figure 6:**
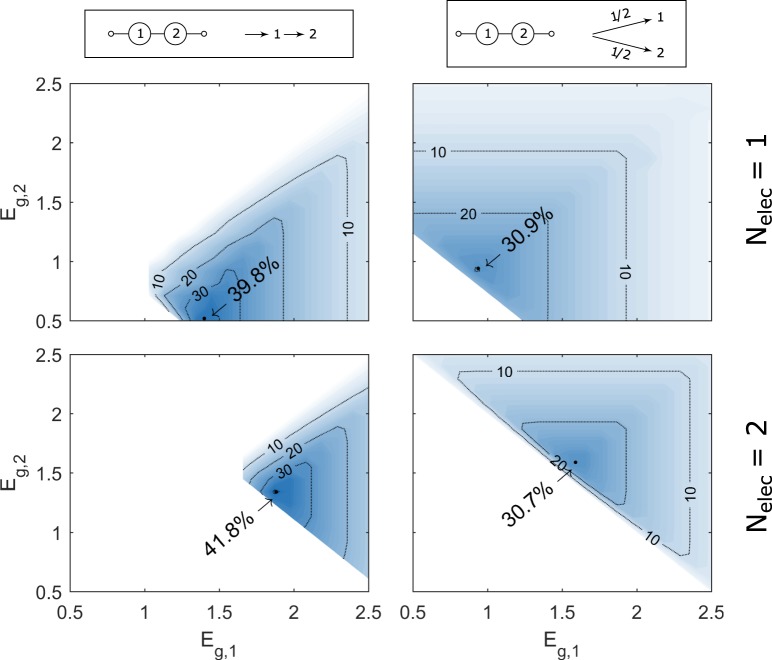
STH Efficiency of various configurations tandem cells, showing optically in series vs in parallel for 1 and 2 electrolysers *N*_*elec*_.

Next, two photo-absorber (*i*.*e* tandem) systems are investigated. Figure 6 shows the efficiencies for tandem systems that are electrically connected in series, but for both optical configurations and various number of electrolysers *N*_*elec*_.

For systems that are optically in series, the second absorber receives light that has not been absorbed by the first absorber and so has passed through. Therefore, the second bandgap must be less than the first (${E}_{g,2} < {E}_{g,1}$) in order for the second absorber to produce a photo-current and for the system to obtain a positive efficiency. The maximum theoretical efficiency is improved marginally by operating with 2 electrolysers and the limiting efficiency at maximum power point for a tandem PV system alone is shown in the supplementary information for comparison (Figure S1).

For the optically in parallel systems shown in Fig. 6, the highest efficiencies are found when ${E}_{g,2}={E}_{g,1}$, as expected. Therefore, only one bandgap needs optimising for optically in parallel systems (and electrically in series) and these diagrams in Fig. 6 can be reduced to the PEC efficiency curves shown in Fig. 4 (*S* = 0.5 and *S* = 1).

### Example 4: Global maximum efficiency for selected permutations of design

**Figure 7:**
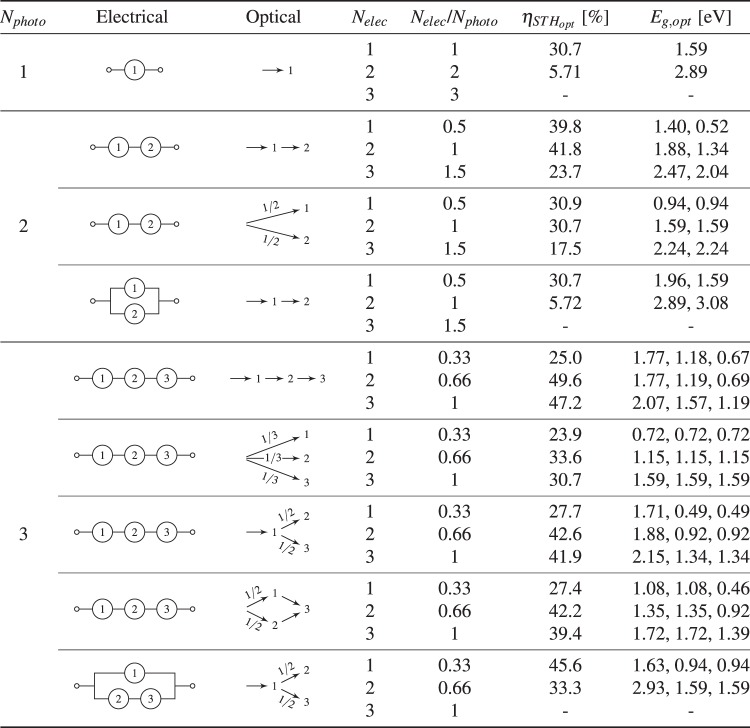
Table of limiting efficiency of selected permutations of design (AM1.5 G, Sun = 1, T = 298.15 K).

For each conceptual design, the bandgaps can be optimised to produce the global limiting efficiency. Mathematically, this is represented as eq. 5 for a particular configuration, light spectrum, temperature *etc*. The results of this can be found in the table in Fig. 7, which outlines the global maximum for the limiting efficiency for various optical and electrical configurations up to *N*_*photo*_ = 3.

5 $${\eta }_{STH,opt}({E}_{g,opt})=\mathop{{\rm{\max }}}\limits_{{E}_{g}\in {\mathbb{R}}}({\eta }_{STH}({E}_{g}))$$

This clearly demonstrates the large variety of designs possible, many of which could obtain high STH efficiencies. Whilst many of these configurations have been studied previously, it can be difficult to compare each subset when different models are used. Therefore, this demonstrates the distinction from previous open access models^12^, in that the code can handle various configurations. This distinction is most apparent when studying systems such as the final entries in Fig. 7, which are similar to the non-monolithic architecture outlined by White *et al*.^29^ and Brillet *et al*.^20^.

## Discussion

This analysis, particularly the table in Fig. 7, will be useful in order to compare experimental efficiencies of various conceptual designs to their respective limiting efficiency. From this, one can determine the realised potential of the device which would allow comparison between different configurations.

For example, the current solar-to-hydrogen efficiency record of 30% is comprised of triple junction cell (in series both electrically and optically) and 2 electrolysers^23^. With bandgaps of 1.895, 1.414, 0.965 eV and a solar concentration of 42 Suns, the maximum theoretical efficiency is 34.3% (assuming ${f}_{g}=2,2,1$, T = 298.15 K and using AM1.5D: direct + circumsolar as this design uses concentration, and ${P}_{solar}=1000$ W m^−2^ as found from integrating AM1.5 G). The global maximum for that configuration, is found at bandgaps of 1.73, 1.17, 0.68 eV and is 45.9%. Therefore, the 30% demonstrates a significant realised potential of that particular configuration.

Whilst this approach is significantly better than assuming a non-radiative model (as explained by Bolton *et al*.^4^), there are a number of simplifying assumptions made. For example, this work does not take into account the built in optical concentration of nanostructured solar cell when compared to planar cells. The code could be modified to account for this by following the work of Xu *et al*.^32^. Furthermore, this analysis currently does not include the realistic performance of the solar cell or electrolysers^8,11^, radiative coupling of sub-cells^8^, semi-transparent upper absorbers^9^, or carrier multiplication^7^. Once again, as the code aims to be extendible, such analysis could be easily incorporated. The authors aim to extend the code so as to be able to include realistic performance losses following the methodologies previously published^8,11,12^.

In conclusion, this paper both extends and unifies previous work on the limiting efficiencies of various PEC architectures and implements this in a freely accessible open-source code.

## Methods

### Model and theory

A comprehensive review of the relevant theory and the mathematical formulation of the model is outlined in full in the supplementary information.

### Numerical implementation and open source code

The analysis was conducted in MATLAB using the inbuilt algorithms to solve systems of non-linear equations (fsolve). A GUI interface was built. Open source code available from http://quicktech.imperialinnovations.co.uk/i/software_apps/Freeware/etaPEC.html under GNU General Public License v3.0.

### Validation of model against literature

**Figure 8:**
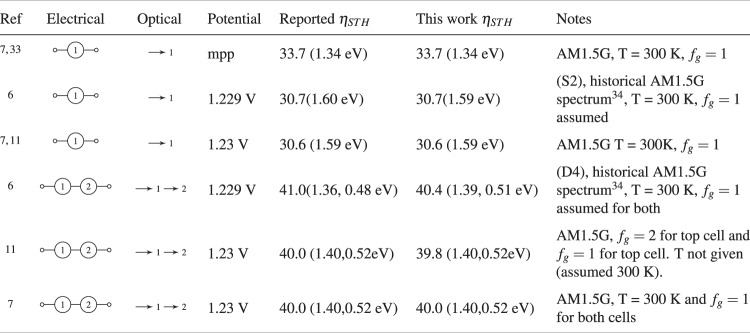
Validation of the model by comparison with existing literature.

As shown in Fig. 8, the results of the script are compared against existing literature^6,7,11,33^ in order to validate the model. Overall, it compares very closely to previous work.

For the comparison with Bolton *et al*.^6^, historical AM1.5 G data was used^34^ as opposed to the more current standard: ASTM G173^22^. However, the results are still slightly different and could be due to different numerical methods used. Interestingly, for the tandem cell case (referred to as D4 in the paper) the authors suggested a second threshold wavelength of 2,610 nm, yet the spectral distribution given by Bird *et al*.^34^ finishes at 2,450 nm. Therefore a second possible reason for any discrepancies here could perhaps arise from a different interpolation method of the solar spectrum data.

We did not compare our data to work by Hu *et al*.^8^ and Seitz *et al*.^9^ as both aimed to model *practical* theoretical limits rather than *fundamental* limits. Furthermore, whilst work by Jacobsson *et al*.^10^ compares well with our analysis, direct comparison is not applicable as it is not a detailed balance approach, but assumes a fixed energy loss due to charge carrier separation of 0.4 eV.

## Electronic supplementary material

Supplementary Information
