# Supplementary material for: A versatile open-source analysis of the limiting efficiency of photo electrochemical water-splitting
Source: Sci Rep. 2018 Aug 24;8:12807. doi: 10.1038/s41598-018-30959-9 (PMC6109057; doi:10.1038/s41598-018-30959-9)
Supplement: Supplementary file 1 — Supplementary Information [file 41598_2018_30959_MOESM1_ESM.pdf]

Supplementary Information for: A versatile open-source analysis of the limiting efficiency of photo electrochemical water-splitting

Isaac Holmes-Gentle[1] **and Klaus Hellgardt**[1,*]

[1]Department of Chemical Engineering, Imperial College London, London, SW7 2AZ, UK.
[*]k.hellgardt@imperial.ac.uk

Contents

Efficiency of tandem PV system **2**
Description of the model **3**
Current voltage relationship of a single photo-absorber . . . 3
Solar driven photolysis . . . 4
Optical configuration . . . 4
Electrical configuration . . . 6
References **9**

Efficiency of series connected tandem PV system

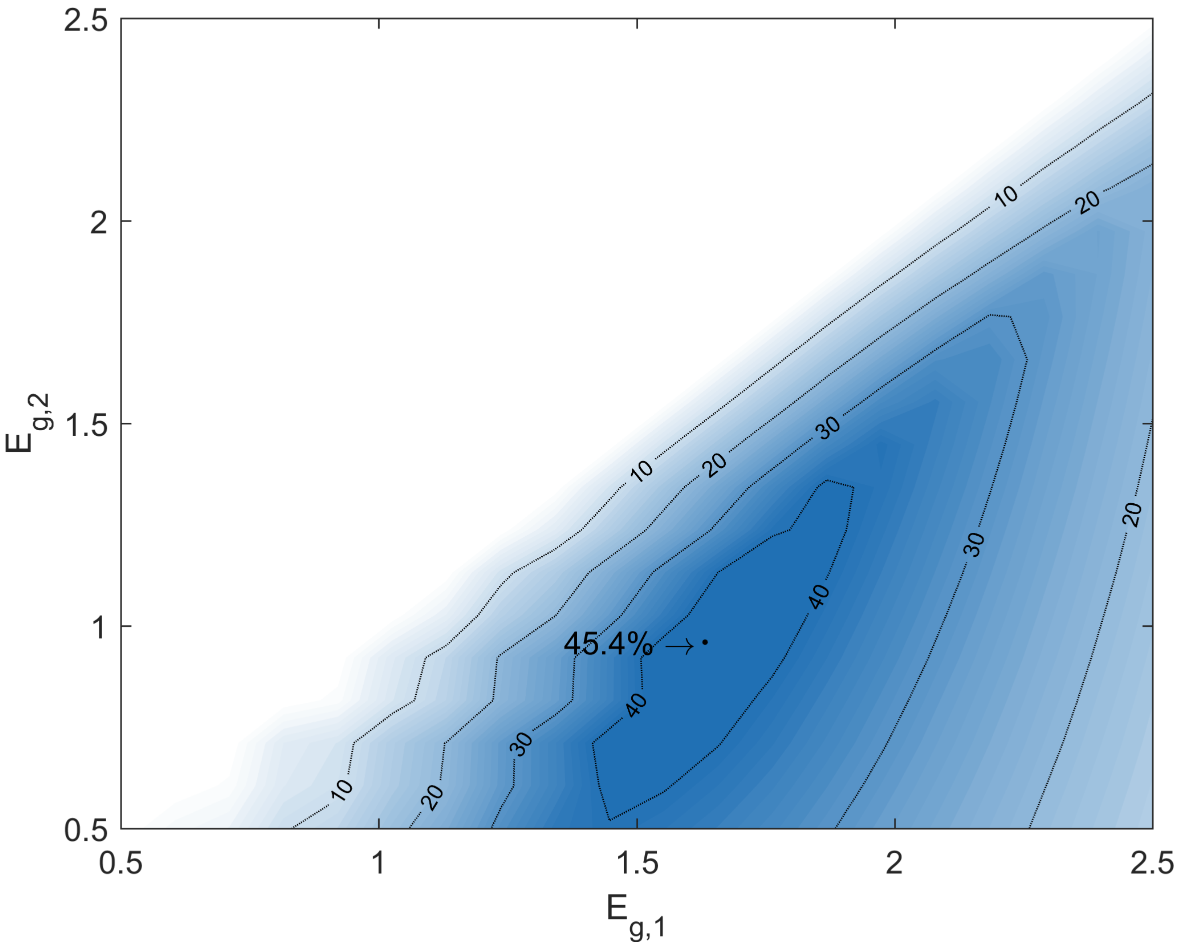

Figure S1. Maximum efficiency of series connected (optically and electrically) tandem photovoltaic system

Description of the model

Current voltage relationship of a single photo-absorber

There are many approaches to determining the limiting efficiency of a solar fuel process as described by Bolton *et al.*[1]. In this work, we approach the analysis from a 'detailed balance' type methodology pioneered by Shockely and Queiser[2]. Following the derivation from various sources[3–5], it is possible to derive the theoretical current-voltage behaviour of a single bandgap absorber cell. Net current density is given by the detailed balance of the current from absorbed photons minus the current lost due to radiative recombination (eq. (1)). Photons absorbed are from the ambient surrounding (radiating blackbody radiation at a temperature of T_a) and from the sun (at a temperature of T_s). Radiative recombination is unavoidable and means such a conversion system cannot exceed the ideal Carnot efficiency limit ($\eta_{carnot} = 1 - T_a/T_s$). Analyses that omit this term will therefore be inaccurate as explained by Bolton *et al.*[1].

$$J = J_{abs} - J_{rad} \tag{1}$$

The generalised form of Planck's radiation law, given in eq. (2), can be used to calculate the ideal blackbody photon emission flux b_i, normal to the surface per energy interval dE. This generalised form includes the effect of the chemical potential $\Delta\mu$ due to excitation separation on the radiated photon flux[5]. For an ideal solar cell where transport is lossless[6], the chemical potential difference generated can be expressed as qV. For the sun and ambient surrounding, $\Delta\mu = 0$. The subscript i denotes the blackbody such as the sun (s), emission from the solar cell (e), the ambient surroundings (a). The geometric factor F_i is equal to π for hemispherical acceptance or emission.

$$b_i(E,\Delta\mu,T) = \frac{2F_i}{h^3c^2}\frac{E^2}{e^{(E-\Delta\mu)/kT}-1} \tag{2}$$

Assuming the ambient and the cell is in thermal equilibrium with its surroundings and that one photon excites only one electron (*i.e* no multiple carrier generation).

$$J_{abs} = q\int_0^\infty \eta_{QE}(E)b_s(E,0,T_s) + \eta_{QE}(E)\left(1-\frac{F_s}{F_e}\right)b_a(E,0,T_a)dE \tag{3}$$

where $b_s(E)$ and $b_a(E)$ is the solar photon flux and ambient photon flux normal to the surface per energy interval dE. If the cell has an abrupt absorption threshold, and that the angular range of the sun is small in comparison to the ambient ($F_s/F_e \approx 0$) we can write this as follows. At thermal equilibrium in the dark, it can be shown that $b_a(E,0,T_a)$ must equal $b_e(E,0,T_a)$ and henceforth the photon flux from ambient is substituted for the photon flux emitted by the solar cell in the dark (*i.e* $\Delta\mu = 0$). And therefore eq. (3) can be written as eq. (4), assuming that all excitations (from absorption of above bandgap photons) that survive radiative recombination are collected.

$$\text{if} \quad \eta_{QE} = \begin{cases} 1, & E \geq E_g \\ 0, & E < E_g \end{cases} \quad \text{then} \quad J_{abs} = q\int_{E_g}^\infty b_s(E,0,T_s) - b_a(E,0,T_a)dE \tag{4}$$

The radiative recombination current under illumination (so $\Delta\mu = qV$) can be similarly written if we assume the cell is a selective blackbody, with an abrupt absorption threshold:

$$J_{rad} = q\int_{E_g}^\infty b_e(E,qV,T_a)dE \tag{5}$$

And therefore, eq. (1) can be expressed as:

$$J = q\int_{E_g}^\infty b_s(E,0,T_s) - (b_e(E,qV,T_a) - b_e(E,0,T_a))dE \tag{6}$$

Equation (7) shows an approximation that can be made for large enough bandgaps (> 0.2 eV) for the radiative recombination of the solar cell under illumination[7,8]. Also, here f_g is introduced where it is the geometric factor that specifies whether the planar plate can radiate from both sides ($f_g = 2$) or just the front side ($f_g = 1$).

$$b_e(E,qV,T_a) = \frac{2f_g\pi}{h^3c^2}\frac{E^2}{e^{(E-qV)/kT_a}-1} \approx \frac{2f_g\pi}{h^3c^2}\left(e^{qV/kT_a}\frac{E^2}{e^{E/kT_a}-1}\right) \tag{7}$$

Therefore we can approximate the ideal current voltage relationship of the solar cell with ideal diode equation:

$$J = J_L - J_o(e^{(qV/kT_a)} - 1) \tag{8}$$

where J_o is given by:

$$J_o = \frac{2f_g q\pi}{h^3c^2}\int_{E_g}^{\infty}\frac{E^2}{e^{E/kT_a}-1}dE \tag{9}$$

The solar spectrum of light can be approximated by a blackbody at a temperature of ~6000 K. In this case, $J_L = q\int_{E_g}^{\infty} b_s(E, 0, 6000K)dE$ and Planck's law, eq. (2), can be used to calculate b_s. Alternatively, a standardised dataset could be used which is a more accurate representation of the terrestrial solar spectrum, such as ASTM G173-03 reference spectra - 37° tilted hemispherical (AM 1.5G)[9] and therefore:

$$J_L = q\int_{E_g}^{\infty} b_{AM1.5G}dE \tag{10}$$

The photovoltaic efficiency can be defined as $\eta_{PV} = \frac{P_{PV}}{P_{solar}}$ where $P_{PV} = J(V)V$. The maximum efficiency will be found at the so called maximum power point at a particular voltage that satisfies $\frac{d(P_{PV})}{dV} = 0$.

Solar driven photolysis

If the photovoltaic device is coupled to an electrochemical process the free energy of the electrons can be stored in the chemical potential of a species produced: *i.e* a fuel such as H_2.

The efficiency of this solar to fuel process can be expressed as:

$$\eta_{STF} = \frac{J(V)\eta_F E_{rxn}}{P_{solar}} \tag{11}$$

Where $J(V)$ is the current provide at some voltage V from the photo-absorber(s), E_{rxn} is the thermodynamic potential (gibbs rather than thermoneutral), $V = E_{rxn} + V_o$. For a ideal cell, the overpotential V_o will be equal to zero. In reality an overpotential is required due to energy lost to entropy in transport and kinetic processes. For solar water splitting, $E_{rxn} = E_{H_2} = 1.229$ V at 300 K. The faradaic efficiency η_F is the efficiency by which charge is transferred to the specific reaction of interest. Henceforth, it is assumed that $\eta_F = 1$.

If the photo-system is driving multiple electrolysis systems that are electrically in series, then the solar to fuel efficiency is given by eq. (12) where i is the specific electrolyser and N_{elec} is the total number of electrolysers.

$$\eta_{STF} = \sum_{i=1}^{N_{elec}}\frac{J_i(V_i)E_{rxn,i}}{P_{solar}} \tag{12}$$

In this work we will only consider electrolysers in series, hence for water splitting the solar to hydrogen efficiency can be written as:

$$\eta_{STH} = \frac{N_{elec}J(V)E_{H_2}}{P_{solar}} \tag{13}$$

Optical configuration

For a single photo-absorber, the light falling on the surface will be the complete input solar spectrum as defined in eq. (10). If there is more than one photo-absorber, consideration has to be made as to how they are optically configured, and Figure S2 shows the different configurations for up to 3 photo-absorbers.

In order to model this, the configuration was represented as a directed acyclic graph made up of nodes and edges which connect nodes together. The first node is the input light spectrum (*e.g* AM1.5G) and each photo-absorber is placed at subsequent nodes and the edges represented the light path. The light before and after the photo-absorber at node i will be denoted by b_i^- and b_i^+ respectively. The light at node 0 (input node) will therefore be $b_0^+ = b_{AM1.5G}$.

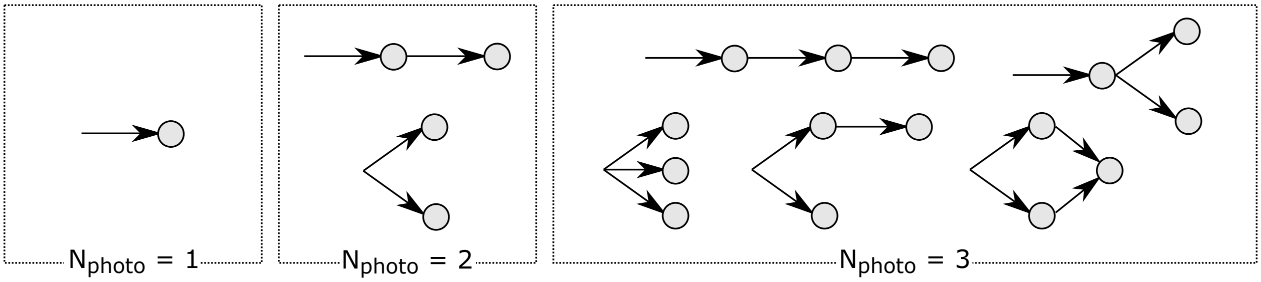

Figure S2. Unique optical configurations for up to three photo-absorbers

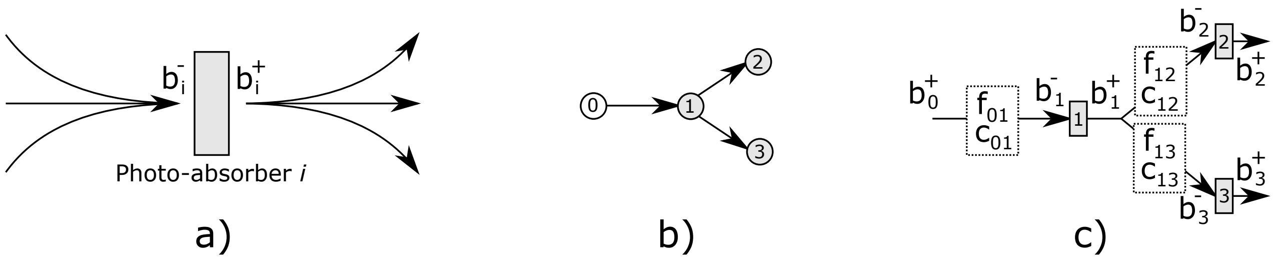

Figure S3. a) Diagram of notation for photo-absorber node i, b) an example optical configuration showing the directed graph c) the same configuration showing the notation for nodes and edges

For each connection between nodes, two quantities are specified. Firstly, the split fraction f_{ij} from node i to j is defined in eq. (14) as the fraction of light reaching j versus the total amount of light leaving i. Here b_{ij} is the photon flux from node i to node j.

$$f_{ij} = \frac{b_{ij} A_j}{b_i^+ A_i} \tag{14}$$

Secondly, the area concentration between nodes is defined in eq. (15). This may differ from the 'light concentration' if the split fraction of that edge is less than unity.

$$c_{ij} = \frac{A_j}{A_i} \tag{15}$$

In order to calculate the light at each node, the topological order of directed acyclic graph was computed as the graph is acyclic. Therefore before the light at a successor node was calculated, all the predecessor nodes had already been calculated. Light at a particular node i is given by the summation of the light from previous nodes, taking into account the transmission $t_i(E)$ of the photo-aborber at node i and the fraction of light from the previous node f_{ij}.

$$b_i^- = \sum_{\forall j} f_{ij} c_{ij} b_j^+ \quad \text{for all predecessor nodes, } j\text{, to node } i \tag{16}$$

$$b_i^+ = t_i(E) b_i^- \tag{17}$$

The transmission function of the photo-absorber at i was assumed to be discontinuous corresponding to a sharp absorption edge.

$$t_i = \begin{cases} 0 & E \geq E_{g,i} \\ 1 & E < E_{g,i} \end{cases} \tag{18}$$

In order to calculate the photocurrent of the photo-absorber corresponding to node i

$$J_{L,i} = q \int_{E_g,i}^{\infty} b_i^- dE \tag{19}$$

Electrical configuration

From electrical connections between the components of the system (photo-absorbers and electrolysers), and the optical configuration of the photo-absorbers, we now can determine the currents and voltages within the circuit.

Multiple electrolysers are considered in series and so the voltage required from the photo-absorber circuit, $V_{tot} = N_{elec}(E_{rxn} + V_o)$ where V_o is the overpotential.

The circuit is represented as a directed cyclic graph where each branch is a component such as a PV or electrolyser. Unfortunately, as MATLAB does not permit multigraphs (multiple edges between nodes), we had to include a third type of branch, which was a simple electrical connection, in order to be able to represent all the permutations of design.

Due to the non-linear nature of the ideal diode equation used for the photo-absorbers, non-linear circuit analysis is required and the 'tableau matrix' methodology described in the book *Linear and Nonlinear circuits* by Chua, Desoer and Kuh[10] was used. Here, n is the number of nodes and b is the number of branches. ϕ_i is the electric potential at node i. j_j and v_j are the branch currents and branch voltage respectively. The electrolyser connects the first (1) and last node (n) and is final branch (b).

1. Construct the directed graph of the circuit and choose the negative side of the electrolyser to be the datum node ($\phi = 0$)
2. Formulate a reduced-incidence matrix, $\boldsymbol{I}$, by removing the row associated with the datum node
3. Apply Kirchoff's current law to each node (except the datum node) ($n-1$ equations)
$$\boldsymbol{I} \cdot \boldsymbol{j} = 0 \tag{20}$$
4. Apply Kirchoff's current law to each node (b equations)
$$\boldsymbol{v} - \boldsymbol{I}^T \cdot \boldsymbol{\phi} = 0 \tag{21}$$
5. Construct the branch equations (b equations)
   - PV equations ($b-1$ equations)
   $$\begin{cases} j_j - J_{PV}(v_j)A_{PV} = 0 & \text{(if PV branch)} \\ v_j = 0 & \text{(if electrical connection branch)} \end{cases} \quad \text{for } j = 1 \text{ to } b-1 \tag{22}$$
   where J_{PV} is the current density of the photo-absorber calculated by eq. (8) and A_{PV} is the illuminated area.
   - Electrolyser equation (1 equation)
   $$v_j - V_{tot} = 0 \quad \text{for } j = b \tag{23}$$

Non-linear root finding methods can be now used on the complete set of equations (KCL, KVL, branch eq.) in order to solve eq. (24).

$$F(\boldsymbol{i}, \boldsymbol{v}, \boldsymbol{\phi}) = 0 \tag{24}$$

As the numerical method used is an iterative one it requires an initial guess for $\boldsymbol{j}$, $\boldsymbol{v}$ and $\boldsymbol{\phi}$. Through trial and error it was found that, whilst perhaps not optimal, the following initial guess appeared to be a relatively robust choice which found the solution in most cases. Furthermore, the fact that the initial guess is calculated rather than supplied by the user, serves to ensure the 'front end' graphical user interface is a simple as possible.

The initial guess for the voltage of the branches is given in eq. (25), and depends whether branch contains a photo-absorber, an electrolyser, or neither.

$$v_{ij} = \begin{cases} v^{mpp} & \text{branch} = \text{PV} \\ 0 & \text{branch} \neq \text{PV} \\ V_{electrolyser} & \text{branch} = \text{electrolyser} \end{cases} \tag{25}$$

The initial guess for the branch current $\boldsymbol{j}$ is given by eq. (26). Here, $max(j^{mpp})$ refers to the maximum current produced by any of the photo-absorbers operating at maximum power point.

$$j_{ij} = \begin{cases} j^{mpp} & \text{branch} = \text{PV} \\ 0 & \text{branch} \neq \text{PV} \\ max(j^{mpp}) & \text{branch} = \text{electrolyser} \end{cases} \tag{26}$$

The initial guess for the potential at each node, $\boldsymbol{\phi}_0$, is calculated by finding the value of $\boldsymbol{\phi}$ that minimises the residual when calculating Kirchhoff's Voltage Law (KVL) using a non-linear least-squares algorithm (see eq. (27) and eq. (21)).

$$\min_{\boldsymbol{\phi}_0 \in \mathbb{R}} \left(\sum_{\forall i} r_i^2 \right) \qquad \text{where} \qquad \boldsymbol{r} = \boldsymbol{v} - \boldsymbol{I}^T \boldsymbol{\phi}_0 \tag{27}$$

Furthermore, in order to aid numerical solution, each quantity with a dimension was scaled before being passed to the numerical solver (fsolve). The scaling factors used were $j^* = \max(J_L)$ and $v^* = e^* = V_{electrolysis}$.

Numerical algorithm flowchart

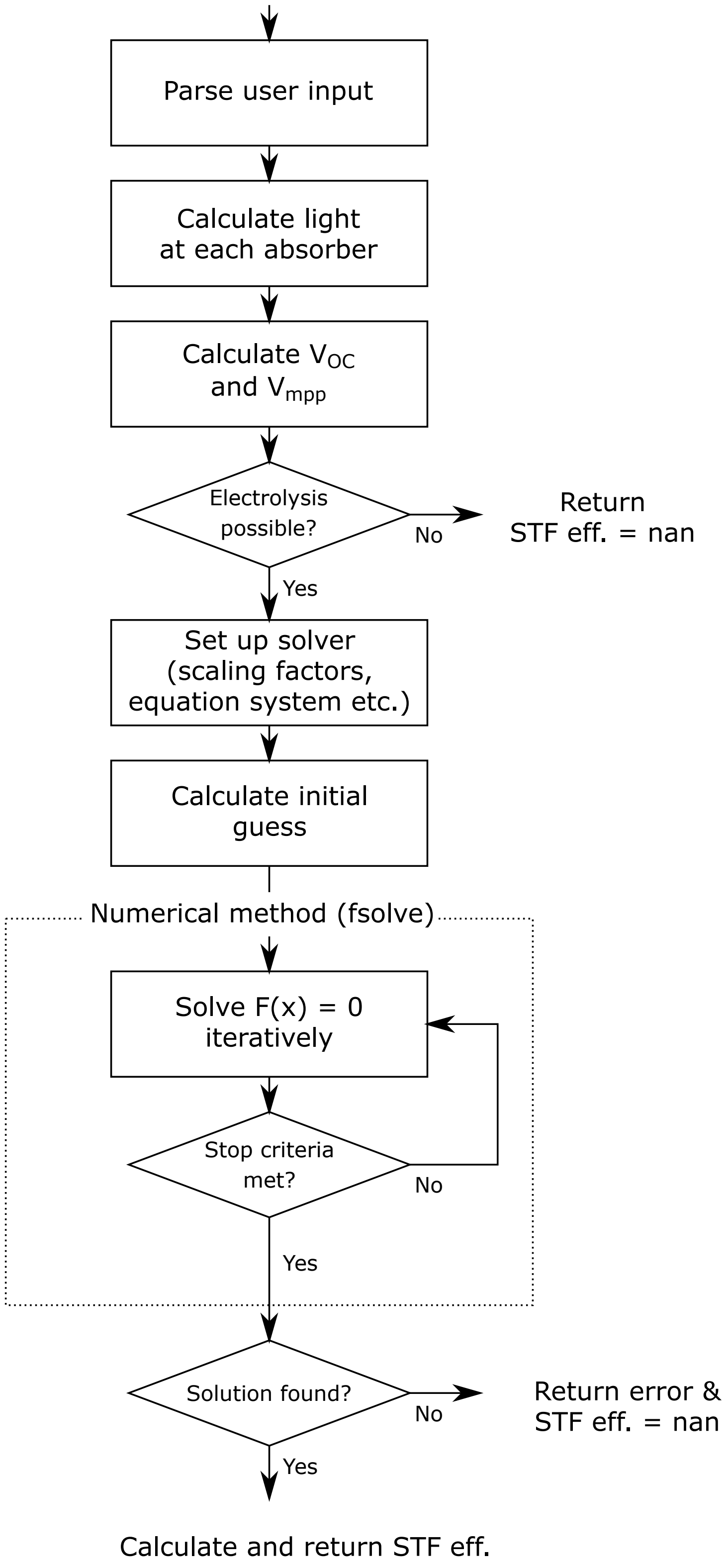

References

1. Bolton, J. R., Haught, A. F. & Ross, R. T. Photochemical energy storage: An analysis of limits. *Photochem. Convers. Storage Sol. Energy* 297–330 (1981).
2. Shockley, W. & Queisser, H. J. Detailed Balance Limit of Efficiency of p-n Junction Solar Cells. *J. Appl. Phys.* **32**, 510 (1961). DOI 10.1063/1.1736034.
3. Hanna, M. C. & Nozik, A. J. Solar conversion efficiency of photovoltaic and photoelectrolysis cells with carrier multiplication absorbers. *J. Appl. Phys.* **100**, 074510 (2006). DOI 10.1063/1.2356795.
4. Hirst, L. C. & Ekins-Daukes, N. J. Fundamental losses in solar cells. *Prog. Photovoltaics: Res. Appl.* **19**, 286–293 (2011). DOI 10.1002/pip.1024.
5. Nelson, J. *The Physics of Solar Cells* (World Scientific Publishing Company, 2003).
6. Araújo, G. L. & Martí, A. Absolute limiting efficiencies for photovoltaic energy conversion. *Sol. Energy Mater. Sol. Cells* **33**, 213–240 (1994).
7. De Vos, A. Detailed balance limit of the efficiency of tandem solar cells. *J. Phys. D: Appl. Phys.* **13**, 839 (1980).
8. De Vos, A. & Pauwels, H. On the thermodynamic limit of photovoltaic energy conversion. *Appl. physics* **25**, 119–125 (1981).
9. G03 Committee. Tables for Reference Solar Spectral Irradiances: Direct Normal and Hemispherical on 37 Tilted Surface. Tech. Rep., ASTM International (2012).
10. Chua, L. O., Desoer, C. A. & Kuh, E. S. *Linear and Nonlinear Circuits* (McGraw-Hill College, 1987).
